# High-Throughput Analysis of Selected Urinary Hydroxy Polycyclic Aromatic Hydrocarbons by an Innovative Automated Solid-Phase Microextraction

**DOI:** 10.3390/molecules23081869

**Published:** 2018-07-26

**Authors:** Stefano Dugheri, Alessandro Bonari, Matteo Gentili, Giovanni Cappelli, Ilenia Pompilio, Costanza Bossi, Giulio Arcangeli, Marcello Campagna, Nicola Mucci

**Affiliations:** 1 Laboratorio di Igiene e Tossicologia Industriale, Azienda Ospedaliero-Universitaria Careggi, Largo P. Palagi 1, 50139 Firenze, Italy; 2 Dipartimento di Medicina Sperimentale e Clinica, Università degli Studi di Firenze, Largo G.A. Brambilla 3, 50139 Firenze, Italy; alessandro.bonari@unifi.it (A.B.); giovanni.cappelli@unifi.it (G.C.); ilenia.pompilio@unifi.it (I.P.); costanza.bossi@unifi.it (C.B.); giulio.arcangeli@unifi.it (G.A.); nicola.mucci@unifi.it (N.M.); 3 Giotto Biotech Srl, Via Madonna del Piano 6, 50019 Sesto Fiorentino (Firenze), Italy; gentili@giottobiotech.com; 4 Dipartimento di Scienze Mediche e Sanità Pubblica, Università di Cagliari, Cittadella Universitaria di Monserrato, SS 554 bivio Sestu, 09042 Monserrato (Cagliari), Italy; mam.campagna@gmail.com

**Keywords:** SPME, OH-PAHs, gas-chromatography, MTBSTFA

## Abstract

High-throughput screening of samples is the strategy of choice to detect occupational exposure biomarkers, yet it requires a user-friendly apparatus that gives relatively prompt results while ensuring high degrees of selectivity, precision, accuracy and automation, particularly in the preparation process. Miniaturization has attracted much attention in analytical chemistry and has driven solvent and sample savings as easier automation, the latter thanks to the introduction on the market of the three axis autosampler. In light of the above, this contribution describes a novel user-friendly solid-phase microextraction (SPME) off- and on-line platform coupled with gas chromatography and triple quadrupole-mass spectrometry to determine urinary metabolites of polycyclic aromatic hydrocarbons 1- and 2-hydroxy-naphthalene, 9-hydroxy-phenanthrene, 1-hydroxy-pyrene, 3- and 9-hydroxy-benzoantracene, and 3-hydroxy-benzo[a]pyrene. In this new procedure, chromatography’s sensitivity is combined with the user-friendliness of *N-tert*-butyldimethylsilyl-*N*-methyltrifluoroacetamide on-fiber SPME derivatization using direct immersion sampling; moreover, specific isotope-labelled internal standards provide quantitative accuracy. The detection limits for the seven OH-PAHs ranged from 0.25 to 4.52 ng/L. Intra-(from 2.5 to 3.0%) and inter-session (from 2.4 to 3.9%) repeatability was also evaluated. This method serves to identify suitable risk-control strategies for occupational hygiene conservation programs.

## 1. Introduction

Polycyclic Aromatic Hydrocarbons (PAHs) are ubiquitous, despite their danger to humans. PAHs can be found in both gaseous and particulate forms. The latter are considered very hazardous to human health; many of the studies on the effects of air pollution have correlated solid aerosols with PAHs with cancer [1]. Outdoor air pollution in both cities and rural areas was estimated to cause 4.2 million premature deaths worldwide in 2016 [2], mainly attributable to airborne particulate matter (PM) [3,4]. Gherardi et al. indicates that 80% of the suspended PM is represented by PAHs [5]. Benzo[a]pyrene (B[a]P) is often used as a marker for total exposure to carcinogenic PAHs, and Ohura et al. [6] reported that B[a]P contribution to the total carcinogenic potential was in the 51–64% range. The Institute of Occupational Medicine [7] estimated that in 2006 in the EU there were 234,000 workers who were potentially exposed to high levels of B[a]P and about seven million to low levels. Recently, Stec et al. revealed that cancer incidence appears to be higher amongst firefighters compared to the general population [8].

Urinary hydroxylated-PAHs (OH-PAHs) have been used as biomarkers to assess total human exposure to PAHs, with 1-hydroxy-pyrene (1-OH-P) as the most commonly used indicator in biomonitoring studies [9]. The Center for Disease Control and Prevention (CDC) developed a OH-PAHs method used for analyzing urinary samples from the National Health and Nutrition Examination Survey, a comprehensive survey that CDC performs annually to assess exposure of the U.S. general population to PAHs [10]. For many years the American Conference of Governmental Industrial Hygienist (ACGIH) had recommended the determination of urinary 1-OH-P as a biomarker to occupational exposure to PAH mixtures, but without any indication of a limit value. Then in 2017, the ACGIH introduced a Biological Exposure Indices (BEI) value of 2.5 μg/L for 1-OH-P, while proposing urinary 3-OH-B[a]P and the sum of 1- and 2-naphthols as non-quantitative markers [11].

The development of analytical methods to identify suitable risk-control strategies for occupational hygiene conservation programs have aroused interest in the scientific community. Most analytical methods have been published to measure urinary OH-PAHs [12,13,14,15,16,17,18,19,20,21,22,23,24,25], which have two to three benzene rings, and only eleven studies [26,27,28,29,30,31,32,33,34,35,36] have considered the determination of OH-PAHs with more than three benzene rings, particularly 3-OH-B[a]P.

The use of MS techniques, particularly gas chromatography (GC) and liquid chromatography (LC), are indispensable tools in metabolomic science owing to their high sensitivity and specificity. In assessing B[a]P exposure—the only PAH classified as category 1 by the International Agency for Research on Cancer—urinary 3-OH-B[a]P determination plays a fundamental role; however the hyphenated chromatographic MS procedures proposed for its analysis are based on age-old methodologies resulting in many manual operations, these have several drawbacks such as uncertainty of the determination and higher overall costs [26,27,29,33,34,35,36]. The use of liquid/liquid extraction (LLE) or SPE with evaporation to dryness of the analyte solution followed by reconstitution in a suitable solvent for injection into the chromatographic system—with or without derivatization—are the typical sequences for monohydroxy PAHs in urine. Currently, four GC methods using *N*-methyl-*N*-(trimethylsilyl) trifluoroacetamide (BSTFA) as a derivatizing agent via extraction with hexane or pentane and related analysis by single [27], QpQ [34], or high-resolution [26,35] MS were proposed. Regarding the LC-triple quadrupole analyses, Raponi et al. [29] and Zangh et al. [36] reported using SPE, while Luo et al. [33] included also reaction with dansyl chloride (DNS). These existing assays have limitations, namely, their complexity, their use of solvents, and the need for clean-up steps to extract and eliminate interfering compounds from the urine; all of those involved lengthy manual operations, bigger costs, uncertainty in the determination analysis, and the possible loss of analytes. For these reasons, simultaneous and more sensitive assay methods were clearly needed.

Miniaturization is increasingly applied in analytical chemistry, resulting in savings in both time and costs throughout the sampling process, typically the most time-consuming and error-prone stage. Specifically, the microextraction via solid phase technique was initially distributed by Supelco (Bellefonte, PA, USA) under the name of Solid Phase MicroExtraction (SPME), [37,38,39,40,41]. At the same time, other companies were also working on devices using the same concept: SPME Arrows [42], MicroExctraction by Packed Sorbent (MEPS) [43], Stir Bar Sorptive Extraction (Twister, SBSE) [44], Solid Phase Dynamic Extraction (Magic Needle, SPDE) [45], In-Tube Extraction (ITEX) [46], HiSorb Sorptive Extraction [47], and Monolithic Material Sorptive Extraction (MonoTrap) [48].

SPME analysis is considered one of the major breakthroughs in 20th-century analytical chemistry; it was the first powerful miniaturized sampling technique developed for GC. SPME, which was invented by Pawliszyn in 1989 [49], integrates sampling, extraction, concentration, and sample introduction into a single step and the extraction requires no polluting organic solvent. Through this, the principles of green chemistry are applied to not only chemical engineering and synthesis, but also increasingly analytical chemistry [10,21,50,51]. Since 2009, significant progress has been achieved by market introduction of the Fast Fit Fiber Assemblies (FFA) [52]. This new generation of SPME fiber was developed by Chromline (Prato, Italy), in cooperation with Supelco, expanding the applicability of SPME; the product line is centered around the SPME FFA barcodes that can be automatically exchanged on a three axis autosampler equipped with the Multi Fiber EXchanger (MFX) system[53].

Therefore, we sought to simplify sample treatment by using the SPME technique in off- and on-line modes for seven OH-PAHs, namely, 1-hydroxy-naphthalene (1-OH-Nap), 2-hydroxy-napthalene (2-OH-Nap), 9-hydroxy-phenanthrene (9-OH-Phen), 1-OH-P, 3-hydroxy-benzoanthracene (3-OH-B[a]A), 9-hydroxybenzo-anthracene (9-OH-B[a]A), and 3-OH-B[a]P. In order to do this, we fully automated the on-line mode, and in the off-line mode we reduced human intervention before GC/triple quadrupole-mass spectrometry (QpQ-MS) analysis. These innovations form a new user-friendly SPME platform which provides relatively prompt results with a high degree of selectivity, precision, and accuracy.

## 2. Results and Discussion

Several OH-PAHs have been suggested as urinary biomarkers to estimate PAH exposure. For instance, 1-OH-P has long been used due to its relatively high levels, even if pyrene is not carcinogenic. However, the profile of PAHs depends on their emission source, and therefore extrapolation of its presence would be imprecise. Conversely, analyzing the hydroxy metabolites of B[a]P and B[a]A would more accurately assess exposure to PAHs. In addition, Gundel et al. [54] proposed 3-OH-B[a]A as an indicator for this, also due to the fact that it is excreted in relatively high concentrations in urine. However, since smoking is a significant source of PAHs it represents a confounding factor. The largest difference between smokers and non-smokers in PAH metabolite concentrations regard 2-OH-Nap, 1-OH-P and OH-phenanthrenes [55,56,57]. Moreover, several authors have shown that urinary OH-fluorene levels are positively correlated with smoking status, particularly 1-OH-fluorene [22,57].

An evaluation of the processing steps of the previously-proposed MS methods including 3-OH-B[a]P [26,27,29,33,34,35,36] revealed five critical phases. First off, phenolic compounds are susceptible to oxidation with consequent loss of OH-PAHs; adding gallic acid (50 µg/mL) prior to the evaporation and derivatization steps effectively inhibits this loss, according to Jacob et al. [9]. Otherwise, Woudneh et al. [35] controlled oxidation by employing 2-mercaptoethanol in a nitrogen atmosphere. Secondly, since OH-PAHs are photodegradable [58], this should be avoided by using amber glassware. A third critical area is the wearing down of the injectors, columns, and GC detectors due to the high amounts of BSTFA injected with conventional sample preparation methods. The fourth point we evaluated is a more rapid and less-solvent-consuming derivatization step by using 1.2-dimethylimidazole-4-sulfonyl (DMISC) instead of DNS; this not only reduced the retention time (RT) by three times, but maintained good quality, as shown in our previous work [59]. Lastly, the seven methods do not allow for full automation.

Accordingly, we developed a method in which the fiber SPME derivatization technique was applied after direct immersion (DI), and then coupled with quantitative determination via GC/QpQ-MS. Here three key aspects behind this choice: SPME extraction, derivatization, and automation.

### 2.1. SPME Extraction

The 85-µm polyacrylate (PA) absortive coating was chosen for sampling such a complex matrix as urine, because the analytes do not compete with each other. Because of the liquid coating’s properties, the extraction obeys the rules of liquid-liquid equilibrium:

n = C_0_V_1_V_2_K/KV_1_ + V_2_, (1)

where K is the partition constant SPME fiber liquid polymeric coating/sample, C_0_ is the initial concentration of the analyte in the aqueous solution, V_1_ and V_2_ are the volumes of the coating and the solution in the equilibrium concentration of the analyte in the urine. Although SPME is an equilibrium extraction, it is not exhaustive.

The equilibrium and kinetics of the OH-PAHs versus SPME fiber with liquid coating were calculated. Table 1 illustrates the physicochemical constants of the seven OH-PAHs obtained by Performs Automated Reasoning in Chemistry (ARChem, Danielsville, GA, USA)—a physicochemical calculator that uses computational algorithms based on their basic chemical structures to foresee a wide variety of reactivity parameters, to anticipate trends in sampling extraction.

Pacenti et al. [60] indicated K_ow_ as a good estimator of K, whose value is often very close to the gas phase partition coefficient/aqueous matrix partition coefficient (K_2_ = K_H_/RT) and to the SPME coating/gas phase partition coefficient (K_1_). K = K_2_·K_1_; consequently, knowing K_1_ and K_2_ values allows you to know in advance whether or not the SPME method is advantageous. The K_H_ value of the OH-PAHs ranges between 8.39 × 10^−8^ atm m^3^/mol for OH-Nap and 4.56 × 10^−10^ atm m^3^/mol for OH-B[a]P, as indicated by Pacenti et al. [60] DI-SPME is efficient for compounds with Henry’s constant lower than 1.75 × 10^−7^ atm m^3^/mol when GC-MS/MS is used.

An excellent SPME extraction sensitivity for the urinary OH-PAHs was achieved by immerging the PA fiber in diluted urine; dilution of the urine with distilled water reduces the sensitivity of the method, but increases the precision and the fiber lifetime. The best results were obtained with DI times up to 30 min with temperature-controlled agitation (60 °C and 500 rpm). To remove any liquid sample remaining on the SPME PA fiber after DI extraction, the fiber was placed for 45 s into an SPME fiber conditioning station set at 100 °C. Moreover, reduction in vial diameter by a factor of 3 resulted in an order of magnitude decrease in extraction time, since t, the average time of diffusion through the aqueous layer is proportional to the square of the migration distance, x, and inversely proportional to D_water_ [61],

t = x^2^/2D_water_, (2)

Hence for high-concentration samples, 2 mL filled to the top, using the same dilution ratio can be used instead of 10 mL amber vials.

### 2.2. SPME Derivatization

**Figure 1:**
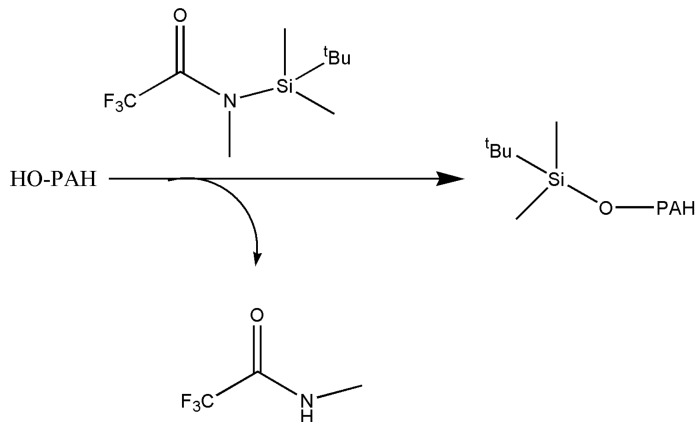
Derivatization of hydroxylated polycyclic aromatic hydrocarbons (OH-PAHs) with *N-tert*-butyldimethylsilyl-*N*-methyltrifluoroacetamide (MTBSTFA).

*N-tert*-butyldimethylsilyl-*N*-methyltrifluoroacetamide (MTBSTFA) as a TBDMS derivatizing agents was used in GC analysis of amino acids and in GC-MS analysis of hydroxylated fluorenes, and it was shown that TBDMS derivatives were thermally stable and had favorable fragmentations upon electron impact (EI) ionization [22,62] (Figure 1).

The MS spectra show two intense fragment ion peaks corresponding to the TBDMS-derivate molecular ion produced by adding 115 Da to the original mass of the compound, and its loss of a tert-butyl fragment of 57 Da. We found that the intensity due to the base peaks of OH-PAHs-TBDMS was about five times higher than that of OH-PAHs-TMS even if the TBDMS derivatives had longer eluting times than the TMS ones.

Next, the effects of time, temperature, and volume of urine and derivatization reagent in automated analysis were evaluated. For on-fiber derivatization low and high values for these three variables (15 and 100 μL MTBSTFA, 25 and 60 °C, and 10 and 60 min) were selected on the basis of previously reported results [63]. The volume of MTBSTFA, the derivatization time, and temperature were fixed at 15 μL, 30 min, and 60 °C, respectively. In order to avoid contamination problems between consecutive samples, on-fiber derivatization was performed in an argon atmosphere in 2 mL silanized amber vials, placed in a 98-position vial tray set to +4 °C.

### 2.3. Automation of the SPME Procedure

**Figure 2:**
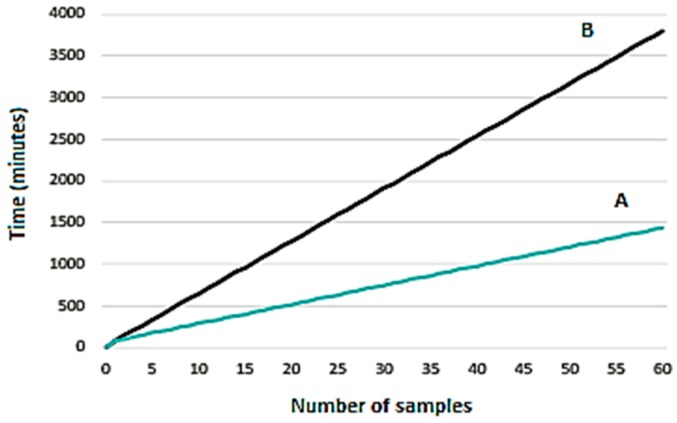
Comparison between solid-phase microextraction fast fit fiber assemblies (SPME-FFA) Multi Off-Line Sampler (**A**) and SPME on-line (**B**) for the analysis of 60 urinary OH-PAHs samples.

New full automation of the procedure was achieved using an *xyz* robotic autosampler coupled with FFA-SPME fibers. In off-line SPME sampling mode by a Multi Off-Line Sampler, the fibers—after the extraction and derivatization steps are manually performed—are placed into the *xyz* autosampler and transported from the MFX 45-position tray to the injector by a SPME holder equipped with a plunger/magnetic system; at the end of the analysis each desorbed fiber is moved back to the tray and the cycle is repeated with a new loaded SPME fiber. Instead, in fully automated on-line SPME mode, the FFA fiber is transported from the vials—containing urine or derivatization agents—to the injector. Figure 2 clearly shows the advantages of using an SPME-FFA Multi Off-Line Sampler calculating a urine extraction time of 30 min, followed by 30 min of MTBSTFA derivatizating reaction plus an analysis time of 20 min; the results are excellent, reducing total analysis time by 2200 min for 60 samples, compared to SPME on-line analysis. The initial economic commitment for the purchase of SPME fibers, as well as for the manual transport steps for extracting and derivatizing, is thus overridden by the off-line method’s high-throughput approach.

To show the above findings the authors present the final results in Table 2.

The resulting calibration curves were linear, in the investigated range for all the OH-PAHs considered, with correlation coefficients >0.99. The precision of the assay (reported as a coefficient of variation, CV%), estimated both as within-session and as inter-session repeatability, resulted in the 2.5–3.0% and 2.4–3.9% range, respectively. Accuracy was within 15% of the theoretical concentration, in line with requirements of the US Food and Drug Administrations for bioanalytical method validation. To demonstrate the applicability of the method to urinary samples, the content of these compounds in non-occupationally-exposed humans, 19 smokers and 21 non-smokers, was analyzed and indicated in Table 3.

## 3. Materials and Methods

### 3.1. Hydrolysis of Conjugated OH-PAHs

Sample processing was conducted in a dark room with limited yellow light. Three mL of urine were spiked with 5 µL of β-Glucuronidase from Helix pomatia (Sigma-Aldrich, Saint Louis, MO, USA, cat. no. G7017-5ML) in 10 mL amber vial (Sigma-Aldrich, Saint Louis, MO, USA, cat. no. 27389). The headspace (HS) over each sample was purged with argon, sealed with screwed caps (Agilent Technologies, St. Clara, CA, USA, cat. no. 8010-1039) and incubated in the dark at 37 °C. After 17 h the samples were diluted with 7 mL of water (10 mL total volume) and doped by deuterated internal standards (ISs) for on- or off-line analysis.

### 3.2. On-Line DI-SPME and xyz Robotic Apparatus

Automated DI-SPME and on-fiber derivatization experiments were carried out by a Flex Autosampler (EST Analytical, Fairfield, CT, USA). The *xyz* robotic system was assembled with devices developed by Chromline (Prato, Italy): a 32-position tray for 10 mL vials, a 98-position tray for 2 mL vials, a Peltier cooler tray (set to 4 °C), a MFX 6-position SPME system, a SPME fiber conditioning station, and agitator. The 10 mL amber vial containing standards/sample was taken automatically from the 32-position tray and was inserted into the agitator, heated (60 °C), and agitated (pulsed agitation, 2 s at 500 rpm and off 4 s). During that period, the FFA-SPME 85-µm PA fiber (Supelco, Bellefonte, PA, USA, cat. no. FFA 57294-U) was immersed directly into the sample solution. After SPME extraction, the fiber was placed for 45 s into an SPME fiber conditioning station set at 100 °C. Subsequently, the SPME on-fiber HS derivatization was performed in the agitator for 30 min at 60 °C, exposing the SPME fiber in 2 mL amber silanized vials (Thermo Fisher Scientific, Waltham, MA, USA, cat. no. MSCERT 5000-S41W) fit with screw thread caps for magnetic transport (Thermo Fisher Scientific, Waltham, MA, USA, cat. no. 9-MSC(BG)-ST101) and containing 15 µL of MTBSTFA (Sigma-Aldrich Saint Louis, MO, USA, cat. no. 394882-10X1ML). Finally, the fiber was inserted into the GC injector equipped with Merlin Microseals (Sigma-Aldrich, Saint Louis, MO, USA, cat. no. 24817-U) for the thermal desorption of the analytes.

### 3.3. Off-Line DI-SPME and xyz Robotic Apparatus

**Figure 3:**
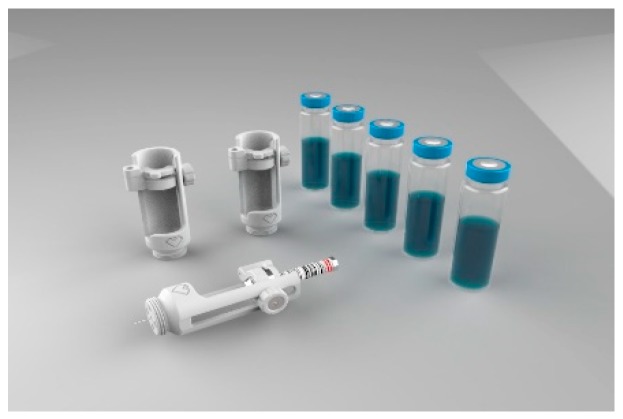
The SPME Multi Off-Line Sampler.

The SPME Multi Off-Line Sampler (Chromline, Prato, Italy) is a holder designed (Figure 3) to be used with FFA SPME fibers; in our case PA 85-μm SPME FFA were used. The holder acts as a support when exposing the SPME fibers in the 10 mL amber vials (60 °C for 30 min), after which they are placed on 15-position magnetic stirrer plates (Chromline, Prato, Italy). After extraction, the FFAs are removed from the Multi Off-Line Sampler and placed for 45 s into an SPME fiber conditioning station set at 100 °C. Subsequently, the SPME on-fiber HS derivatization (30 min at 60 °C) was performed in 2 mL amber silanized vials, placed into the SPME Multi Off-Line Sampler. For desorption the fiber was put into a MFX 45-position SPME system installed on the Flex autosampler coupled with GC instrumentation.

### 3.4. GC/QpQ-MS

Analyses were performed with a Varian 3900 GC equipped with electronic flow control and a Varian 320-QpQ-MS (Agilent Technologies, St. Clara, CA, USA) detector (Table 4).

**Figure 4:**
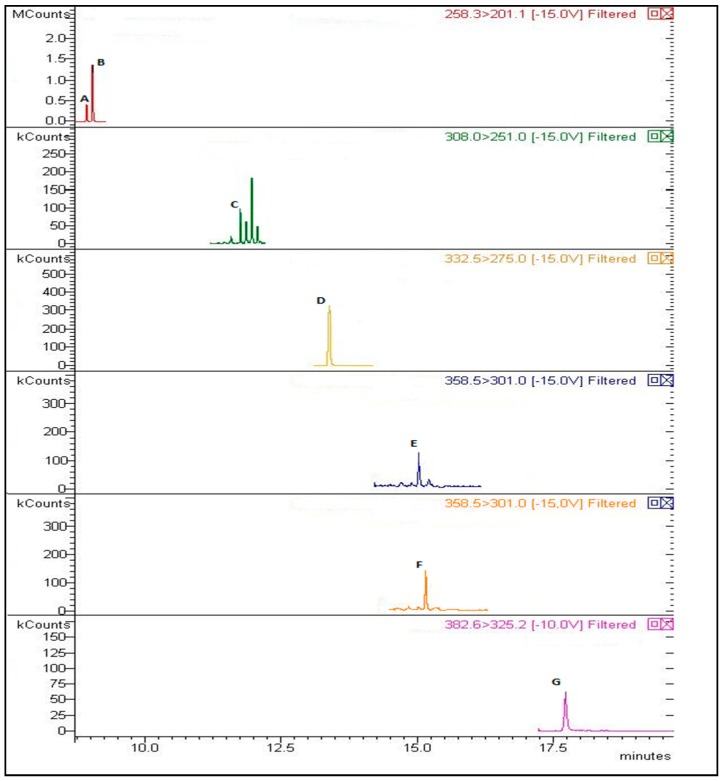
Chromatogram of OH-PAHs by gas chromatography (GC)/triple quadrupole-mass spectrometry (QpQ-MS) in spiked artificial urine. A = 1-OH-Nap (200 ng/L); B = 2-OH-Nap (600 ng/L); C = 9-OH-Phen (50 ng/L); D = 1-OH-P (400 ng/L); E = 3-OH-B[a]A (1 ng/L); F = 9-OH-B[a]A (1 ng/L); G = 3-OH-B[p]A (5 ng/L).

A VF-5 ms + 10 m EZ-Guard fused silica capillary column (internal diameter 0.25 mm, length 30 m and film thickness 0.25 μm) (Agilent Technologies, St. Clara, CA, USA, cat. no. CP9013) was used (Figure 4). For desorbing the analytes, the SPME fiber was introduced into the 1177 Varian GC injector port. A connection with the Laboratory Information Management System (Bika Lab System, Pringle Bay, South Africa) provides a user-programmable suite of options.

### 3.5. Synthesis

1-OH-Nap (cat. no. N1000), 2-OH-Nap (cat. no. 185507), 9-OH-Phen (cat. no. 211281), and 1-OH-P (cat. no. 361518) were purchased from Sigma-Aldrich (Saint Louis, MO, USA). As described by Xu et al, 3-OH-B[a]P was synthesized [64], while 3-OH-B[a]A, and 9-OH-B[a]A were prepared following McCourt’s procedure [65]. The deuterated compounds 1-hydroxy-naphthalene-D7, 2-hydroxy-napthalene-D7, 9-hydroxy-phenanthrene-D9,1-hydroxypyrene-D9, 3-hydroxy-benzoanthracene-D11, 9-hydroxybenzo-anthracene-D11, and 3-hydroxy-benzo[a]pyrene-D11 were prepared by perdeuteration of the unlabeled starting material under the conditions described by Duttwyler et al. [66]; in all cases two reaction cycles were enough to reach a deuteration of above 98%.

### 3.6. Calibration and Method Validation

Spiked artificial urine [12] (1, 2, 4, 8, 16, 32 ng/L) was analyzed and five replicates of each sample were performed to calculate their limits of detection (LOD). A linear regression plot was generated; the LOD is reported as [(YB + 3SB)/m], where Y B is the intercept, SB is its standard deviation, and m is the plot slope. The limit of quantification (LOQ) was then calculated in the same fashion, using 10SB, which corresponds to 3.3 LOD. Samples were processed by calibration curves set as follow: (a) 100, 200, 400, 800, 1600, 3200, 6400 ng/L for 1-OH-Nap and 2-OH-Nap (b) 10, 20, 40, 80, 160, 320, 640 ng/L for 1-OH-P (c) 0.5, 1.0, 2.0, 4.0, 8.0, 16.0 ng/L for 3-OH-B[a]A, 9-OH-B[a]A and 3-OH-B[a]P. The precision of the assay (as a coefficient of variation, CV%) was based on both within-session and inter-session repeatability. Accuracy was evaluated by the recoveries (calculated from the percentage ratio between the measured and the nominal concentration solutions) at all concentrations used for the calibration plot and from certified analytical standards for 1-OH-P (Chromsystems Instruments & Chemicals GmbH, Gräfelfing, Germany, cat. no. 53003). Values of accuracy were then compared with the requirements of the US Food and Drug Administration for analytical method validation. Level quality control samples were prepared and processed in every analytical session from a fresh solution with the IPA with ISs to ensure the precision validity of reported results.

### 3.7. Evaluation of Processing Steps of Previously Proposed MS Methods Including 3-OH-B[a]P

We found five critical processing steps in the seven above-indicated methods [26,27,29,33,34,35,36]: (i) susceptibility to oxidation during evaporation and derivatization, (ii) photodegradation, (iii) BSTFA contamination, (iv) excessive analysis time using DNS, and (v) undue handling time.

## 4. Conclusions

Occupational studies indicate that there is a correlation between PAH exposure and cancer incidence for various human tissues such as lungs, skin, and bladder. As a result, a regular control of the concentrations in the workplace and in life environments trough measuring their metabolites has become mandatory. PAH metabolites in human urine can be used as biomarkers of internal dose to assess recent exposure to PAHs. In previous studies, the oft-reported use of solvent and/or clean-up steps were necessary to extract and eliminate most of the interfering compounds from the urine. But these laboratories used techniques based on age-old methodologies with a low level of automation in which each step represents additional time and potential source of error. Instead, a straightforward, optimized sample preparation strategy minimizes the number of steps.

Our data suggests that automated SPME extraction coupled with GC/QpQ-MS is a viable alternative for OH-PAH analyses. Customized and automatized MS systems for high-throughput screening are not only user-friendly, but they reduce the costs of monitoring occupational health hazards. New sample preparation techniques are currently being increasingly explored because of the considerable need for the automating of sample preparation, and for integrating data management into the analytical process.

## Figures and Tables

**Table 1:** Physical properties and partition coefficients of OH-PAHs evaluated using SPARC. (M.W. = molecular weight; T_eb_ = boiling point; D_water_ = diffusion coefficient of the analyte in water; K_H_ = Henry’s constant; K_ow_ = octanol-water partition coefficient; P_vap_ = vapour pressure).

SMILES strings	M.W. (g/mol)	T_eb_ (°C)	D_water_ (cm^2^/s)	K_H_ (atm/(mol/m^3^))	K_OW_ (Log)	P_vap_ (log(atm))
OC1=CC=CC2=CC=CC=C21	144	269.7	8.08 × 10^−6^	8.39 × 10^−8^	3.04	−6.0
OC1=CC2=CC=CC=C2C=C1	144	269.8	8.08 × 10^−6^	9.10 × 10^−8^	3.11	−6.14
OC1=CC2=C(C3=C1C=CC=C3)C=CC=C2	194	378.9	6.92 × 10^−6^	6.93 × 10^−9^	4.49	−8.67
OC1=CC=C(C=C2)C3=C1C=CC4=CC=CC2=C34	218	454.6	6.41 × 10^−6^	6.37 × 10^−9^	5.01	−8.9
OC(C=C1)=CC2=C1C3=CC4=CC=CC=C4C=C3C=C2	244	537.2	6.15 × 10^−6^	3.24 × 10^−10^	5.71	−11.86
OC1=CC=C2C(C=C(C=CC3=C4C=CC=C3)C4=C2)=C1	244	537.2	6.15 × 10^−6^	3.21 × 10^−10^	5.71	−11.86
OC1=CC=C(C=C2)C3=C1C=CC4=CC5=CC=CC=C5C2=C43	268	564.5	5.74 × 10^−6^	4.56 × 10^−10^	6.28	-11.81

**Table 2:** Limits of detection (LOD), limit of quantification (LOQ), accuracy and precision for each OH-PAH measured in urine samples.

Response factor Plot and Limit of Detection and Quantification								
		**1-OH-Nap**	**2-OH-Nap**	**9-OH-Phen**	**1-OH-P**	**3-OH-B[a]A**	**9-OH-B[a]A**	**3-OH-B[a]P**
Least-squares linear regression parameters	m	1.0924	1.0922	1.1125	1.1126	1.1244	1.1240	1.1245
	b	0.1893	0.2204	0.0769	0.0828	0.0455	0.0453	0.0368
Coefficient of Correlation		0.99	0.99	1.00	0.99	0.99	1.00	1.00
LOD (ng L^−1^)		4.52	4,03	2.53	1.89	0.31	0.33	0.25
LOQ (ng L^−^^1^)		14.91	13.20	8.34	6.23	1.02	1.08	0.82
**Accuracy and precision (%)**								
Within-session accuracy		10.0	9.3	10.3	9.8	10.5	10.2	10.7
Within-session repeatability		2.7	2.7	3.0	2.7	2.5	2.6	2.5
Inter-session repeatability		3.0	3.0	3.6	3.9	3.4	2.4	3.4

**Table 3:** OH-PAHs in human urine of smokers and non-smokers. (SD = standard deviation).

	Non-Smokers	Smokers
	Average (ng/L) ± SD (min-max Value)	Average (ng/L) ± SD (min-max Value)
1-OH-Nap	1040.6 ± 340.7 (150.3–1500.2)	2966.6 ± 904.3 (240.1–3500.6)
2-OH-Nap	1879.2 ± 402.4 (201.6–2001.3)	4297.5 ± 1151.2 (2898.3–8214.4)
9-OH-Phen	<LOD ± 0.54 (<LOD–3.2)	<LOD ± 0.66 (<LOD–3.6)
1-OH-P	59.3 ± 27.4 (25.1–166.7)	291.4 ± 89.3 (178.0–647.2)
3-OH-B[a]A	0.43 ± 0.21 (<LOD–1.2)	0.60 ± 0.23 (<LOD–1.6)
9-OH-B[a]A	<LOD ± 0.25 (<LOD–1.41)	1.44 ± 0.59 (<LOD–2.3)
3-OH-B[a]P	<LOD ± 0.17 (<LOD–0.91)	0.98 ± 0.14 (<LOD–1.32)

**Table 4:** GC/QpQ-MS method parameters.

**GC Conditions**	
Injection	300 °C, 20:1 split mode. Liner 0.75 mm i.d.
Oven	80 °C (1 min) increased to 20 °C/min end to 320 °C (5 min)
Column flow	Helium (99.999%) at a flow rate of 1.2 mL/min
Retention time	1-OH-Nap (8.90 min); 2-OH-Nap (9.05 min); 9-OH-Phen (11.96 min); 1-OH-P (13.38 min); 3-OH-B[a]A (14.93 min); 9-OH-B[a]A (14.93 min); 3-OH-B[a]P (17.72 min)
GC interface	280 °C
**MS Parameters**	
Mode	EI
Filament	Electron energy, 70 eV. Filament current 50 μA
Source	Temperature, 200 °C. Pressure, 8 Torr.
Collision gas	CID gas, Argon. CID gas pressure, 2.00 mTorr
Collision energy	1-OH-Nap 15 eV; 2-OH-Nap 15 eV; 9-OH-Phen 15 eV; 1-OH-P 15 eV; 3-OH-B[a]A 15 eV; OH-9-B[a]A 15 eV; 3-OH-B[a]P 10 eV.
**SRM Transition**	
1-OH-Nap	Fragment Q1 > Q3 Quantification *m/z* 258.5→201.2 Confirmation *m/z* 201.4→185.0
2-OH-Nap	Q1 > Q3 258.5→201.2 201.4→185.0
9-OH-Phen	Q1 > Q3 308.5→251.2 251.4→235.0
1-OH-P	Q1 > Q3 332.5→275.0 275.4→259.0
3-OH-B[a]A	Q1 > Q3 358.5→301.1 301.5→285.0
9-OH-B[a]A	Q1 > Q3 358.5→301.1 301.5→285.0
3-OH-B[a]P	Q1 > Q3 382.6→325.2 382.6→309.6
